# The Case for Breast Implant Removal or Replacement Without Capsulectomy

**DOI:** 10.1007/s00266-020-02079-1

**Published:** 2021-02-11

**Authors:** Eric Swanson

**Affiliations:** Swanson Center, 11413 Ash St, Leawood, KS 66211 USA

*Level of Evidence V* This journal requires that authors assign a level of evidence to each article. For a full description of these Evidence-Based Medicine ratings, please refer to the Table of Contents or the online Instructions to Authors www.springer.com/00266.

The link between textured implants and Breast Implant-Associated Anaplastic Large-Cell Lymphoma (BIA-ALCL) [1, 2] has led many women to consider replacement of their breast implants with smooth devices. Many women today are considering explantation because of concerns regarding Breast Implant Illness (BII). Capsulectomy is widely recommended [3].

Surgeons’ opinions differ regarding the value of implant replacement. Some surgeons advise their patients that the risks of reoperation may exceed the risk of BIA-ALCL, and recommend against implant replacement in asymptomatic women [3]. The U.S. Food and Drug Administration has not recommended that patients replace their textured breast implants [2], but has not recommended that they keep them either.

Historically, the risk of BIA-ALCL has been considered very low, but this risk estimate has increased dramatically in recent years. In 2011, the estimated risk was 1:500,000 women/year [4]. In 2017, the calculated lifetime prevalence was 1:30,000 in women with textured breast implants [5]. In 2020, Cordeiro et al. [4] estimated the cumulative risk over 20 years as 1:100 among breast reconstruction patients implanted with Biocell devices (Allergan, recently acquired by AbbVie, Lake Bluff, Ill.). Hall-Findlay [6] has diagnosed 2 cases of BIA-ALCL among approximately 100 cosmetic breast augmentation patients (1:50) she implanted with Biocell devices [6].

When considering the pros and cons of implant replacement surgery, an important consideration is whether a capsulectomy is recommended [3]. A capsulotomy with implant replacement is a short operation with very low morbidity [3, 7]. The anesthetic risk is minuscule. A surgical death has not been reported [3]. By contrast, a capsulectomy is often a difficult, time-consuming operation with much greater morbidity [3]. It is often impossible to entirely remove the capsule, especially in women with subpectoral implants, begging the question regarding the need for a capsulectomy in the first place if at least some of the capsule is left in situ. The surgeon risks more bleeding, pneumothorax, nerve injury, and skin loss [3]. Efforts to remove the axillary portion of a capsule risk injury to the brachial plexus and axillary vessels [3, 8, 9]. Injury to the axillary vein and at least one death from capsulectomy have occurred [3].

Despite the additional risks, some surgeons go to great lengths to strip the capsule off the chest wall, dissecting through intercostal muscles, and even taking video of the procedure to document its complete removal [10]. Plastic surgeons advertise their expertise on internet sites such as enblocsurgeons.com [11]. Some surgeons perform hundreds of explantations with capsulectomies annually [10, 12]. One plastic surgeon’s practice is exclusively devoted to explantation, “en bloc” capsulectomy, and mastopexy [10]. All patients undergo “en bloc” capsulectomy. No patients are offered replacement implants [10].

Because the physical basis (if one exists) for BII is unknown, and does not depend on a diagnostic test, the eligibility criteria for surgery are loose. Patients who have noticed they are getting fatigued easier or experiencing more headaches are candidates. Any woman who is anxious about having BII is a candidate. The patient is frequently left with a deflated, scarred breast, and may resemble a bilateral breast reconstruction candidate. A major motivation for women to have breast implants is to improve their self-image; these patients are left with the opposite outcome. Women who find that their ailments are not relieved after explantation may wish to have new implants inserted. This option should be available to them.

The capsule, with its associated biofilm, has been implicated as a causative factor in recurrent capsular contracture, BIA-ALCL, and BII [3, 13]. However, capsulectomy has never been shown to provide a systemic benefit to patients [3, 8]. Plastic surgeons may be surprised to learn that no evidence exists that capsule removal at the time of implant replacement reduces the risk of a subsequent BIA-ALCL diagnosis [3, 14]. Similarly, there is no scientific support for a capsulectomy to reduce the risk of BII [3, 15]. Capsulectomy is not consistently followed by disease remission [3, 16].

Studies have been published attesting to the post-explantation relief of the myriad symptoms comprising BII [12, 13, 16]. A recent study reported a highly significant (*p* < 0.0001) improvement in symptoms in 11 different categories [12]. One of these categories is breathing problems. The authors speculate that removing the capsule frees up the rib cage to expand easier. It is difficult to conceive of a physical explanation for a uniformly dramatic improvement in every physical condition evaluated. This is a clue to the psychological effect. Many women who elect (or rather are influenced) to have expensive, painful surgery are inclined to report a postoperative improvement. To do otherwise would create cognitive dissonance. In addition, these women typically have their implants and capsules removed together, so that it is impossible to isolate any possible health benefit from the capsulectomy.

Although the conventional wisdom supports capsulectomy to reduce the risk of recurrent capsular contracture, even this assumption is open to question [3, 7]. A capsulectomy leaves a large internal wound, which heals with the formation of a new capsule. One capsule is replaced by another. According to the infection theory, removing the old capsule also removes old, infected biofilm. However, a capsule is not a microbiological barrier [3]. This procedure cannot be expected to sterilize the wound. The new capsule, and new biofilm, will form in the same micro-environment, exposed to the same commensal bacteria, as the original capsule [3]. A capsulectomy, along with a site change and implant replacement, has not proven to be an effective solution for capsular contractures, with recurrence rates as high as 53% [17]. By comparison, the recurrence rate after capsulotomy alone is 22.7%, and even lower, 13.6%, in women with intact implants [7].

A capsule with suspicion or evidence of pathology is different. In this situation, there is universal agreement that the capsule should be removed [3, 18]. Such surgery is needed to ensure that a BIA-ALCL tumor is removed, and also to provide a specimen for examination. It may be necessary to remove adjacent tissue simultaneously, the real definition of “en bloc.” [14, 19]

Today, patients receive most of their information on the internet. Some surgeons (inaccurately) [19] advertise “en bloc” capsulectomies as the proper treatment for BII [11]. Patients request this operation. Many surgeons accommodate this request and send the capsule for pathologic examination [9]. One may question the logic in recommending against implant replacement in asymptomatic patients with textured implants who are concerned about BIA-ALCL, but then insisting on capsulectomy, and pathologic examination, if the patient decides to have her implants replaced anyway. If the capsulectomy and pathologic examination are mandatory, so is the surgery [3].

Any recommendation regarding capsule treatment is limited by the present deficiency in knowledge regarding the underlying disease process for BIA-ALCL, BII, and capsular contracture. Few studies compare the long-term outcomes of capsulotomy versus capsulectomy [7]. It is appropriate, and expected, for surgeons to inform patients regarding treatment alternatives. Less traumatic options are usually preferred if there is no clear advantage for more aggressive surgery. In the author’s practice, asymptomatic women are counseled that a capsulectomy provides no known benefit and introduces additional risk and morbidity. The surgeon does not need to perform a capsulectomy just because the patient requests it. No doubt some women seek this operation elsewhere. However, others choose to undergo implant removal or replacement without a capsulectomy (Fig. 1, see Video). This choice is certainly a reasonable one.

**Fig. 1:**
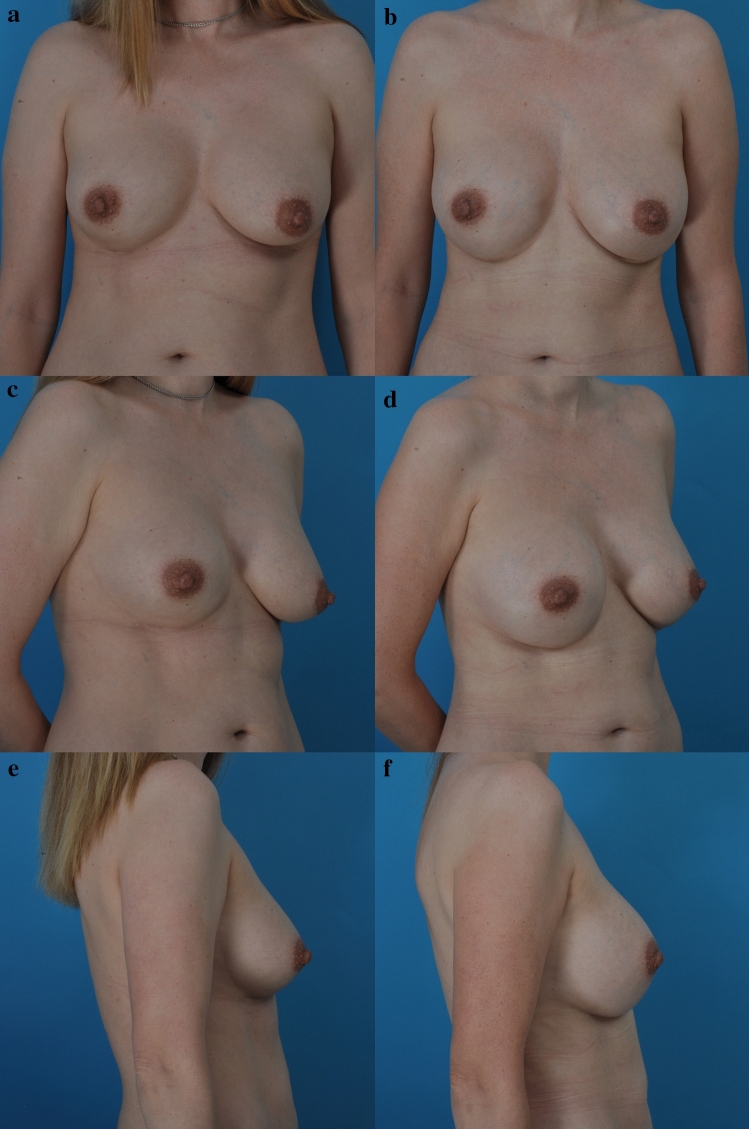
**a**, **c**, **e** This asymptomatic 38-year-old woman is shown before and **b**, **d**, **f** 6 weeks after replacement of her 290 ml Allergan Biocell textured silicone gel breast implants. She underwent open capsulotomies and insertion of new Allergan 405 ml round, smooth, moderate profile silicone gel implants. The same subpectoral pocket was used. She also underwent liposuction of her abdomen and flanks

## Supplementary Information

Below is the link to the electronic supplementary material.

This 38-year-old woman received Allergan Biocell implants at the time of her breast augmentation 8 years previously, performed elsewhere. Although she was asymptomatic, she was concerned regarding her future risk of BIA-ALCL. The preoperative evaluation, surgery, and examination 24 hour after surgery are provided. (MP4 90093 kb)
